# Total Phenolics and Total Flavonoids Contents and Hypnotic Effect in Mice of *Ziziphus mauritiana* Lam. Seed Extract

**DOI:** 10.1155/2013/835854

**Published:** 2013-06-04

**Authors:** Aye Moh Moh San, Suchitra Thongpraditchote, Pongtip Sithisarn, Wandee Gritsanapan

**Affiliations:** ^1^Department of Pharmacognosy, Faculty of Pharmacy, Mahidol University, 447 Sri-Ayudthaya Road, Ratchathevi, Bangkok 10400, Thailand; ^2^Department of Physiology, Faculty of Pharmacy, Mahidol University, 447 Sri-Ayudthaya Road, Ratchathevi, Bangkok 10400, Thailand

## Abstract

The seeds of *Ziziphus mauritiana* Lam. have been traditionally used for treatment of various complications including insomnia and anxiety. They are popularly used as sedative and hypnotic drugs in China, Korea, Myanmar, Vietnam, and other Asian countries. However, no scientific proof on hypnotic activity of *Z. mauritiana* seeds (ZMS) was reported. In this study, the hypnotic activity of 50% ethanolic extract from ZMS was observed on the loss of righting reflex in mice using pentobarbital-induced sleep mice method. The contents of total phenolics and total flavonoids in the extract were also determined. The results showed that the 50% ethanolic extract from ZMS contained total phenolics 27.62 ± 1.43 mg gallic acid equivalent (GAE)/g extract and total flavonoids 0.74 ± 0.03 mg quercetin equivalent (QE)/g extract. Oral administration of the extract at the dose of 200 mg/kg significantly increased the sleeping time in mice intraperitoneally administered with sodium pentobarbital (50 mg/kg body weight). These results supported the traditional use of ZMS for the treatment of insomnia. The seeds of *Z. mauritiana* should be further developed as an alternative sedative and/or hypnotic product.

## 1. Introduction

*Ziziphus* is a genus in the family Rhamnaceae which contains about 40 species. Fruits and seeds of *Ziziphus *have been widely used as traditional medicines since ancient time. *Z. mauritiana* Lam. is a tropical or subtropical fruit tree widely distributed in many Asian countries such as Afghanistan, Bhutan, India, Indonesia, Malaysia, Myanmar, Nepal, Sri Lanka, Vietnam, Africa, Australia, and Thailand [1]. The seeds of *Z. mauritiana*, a species close to *Z. jujuba *Mill., have been reported as anticancer, antidiabetic, and hypoglycemic agents [2, 3] while the seeds of* Z. jujuba*, which are known as jujube or Chinese date, are popularly, used to treat insomnia and reduce the body temperature and sweat [4, 5]. *Z. mauritiana *seeds have been also used as sedative and hypnotic drugs in many Asian countries. *Ziziphus* seeds contain large amounts of fatty oil and proteins, sterols, and triterpenoid compounds (betulin and betulinic acid) and also contain a large amount of vitamin C [6]. 

Sleep is one of the most deeply healing and revitalizing known experiences. Insomnia, a common problem, is a lack of healthful and restful sleep. Thirty to fifty percent of the populations are reported to be affected by insomnia while 10% of them have chronic insomnia. Fewer than 15% of patients with insomnia receive treatment [7]. Most patients are engaged in long-term use of benzodiazepines (BZDs) analogs to treat insomnia. There are some deficiencies on impaired cognitive function, memory, and general daytime performance in patients treated with these drugs. Moreover, tolerance and dependence are the obvious side effects of the drug's long-term administration [8]. Natural sleep aids, which contain specific constituents of foods and herbal plants, have recently become popular as alternatives to prescription sedative-hypnotics to improve sleep quality and avoid side effects [9]. Therefore, there has been a growing demand for a new class of food constituents and natural products with hypnotic effects. GABAergic neurotransmission plays a key role in sleeping regulation and the BZD-binding site on the GABA-A receptor which is a target for the most sedative-hypnotics [10]. It has been widely reported that polyphenols and flavonoids have sedative-hypnotic effects based on positive allosteric modulation of GABA-A receptors [11]. ZMS have been popularly used as sedative and hypnotic drugs in many Asian countries without any scientific report on hypnotic activity. Our study was aimed to explore the hypnotic activity of 50% ethanolic extract from ZMS, which was previously found to promote high radical scavenging activity [12] through the loss of righting reflex on pentobarbital-induced sleep mice. The contents of total phenolics and total flavonoids in the extract were also determined.

## 2. Materials and Methods

### 2.1. Plant Material

Dried ZMS was purchased from Kaung Su Aung Co., Ltd. Herbs Plantation in Myanmar in June, 2010. The sample was identified by comparison with the voucher specimens at the Bangkok Herbarium, Botanical Section, Botany and Weed Science Division, Department of Agriculture, Bangkok. The voucher specimen (ZM0610) was deposited at Department of Pharmacognosy, Faculty of Pharmacy, Mahidol University, Bangkok, Thailand. The seeds were dried at 55°C for 6 hours and ground with an electronic mill to give moderate powder. The powdered sample was kept in an air tight container protected from light in a cool place until extracted.

### 2.2. Preparation of 50% Ethanolic Extract from ZMS

According to our former report the soxhlet extraction with 50% ethanol was the appropriate extraction method and solvent to promote the ZMS extract with the strongest antioxidant activity [12]. Therefore, the 50% ethanolic extract was prepared from ZMS by this procedure. The powdered sample was placed into a thimble in a soxhlet apparatus and was extracted with 50% ethanol (1 : 35, w : v) at 55°C until exhaust (15 hours). The extract was filtered through a Whatman No. 1 filter paper and the filtrate was concentrated under reduced pressure at 50°C using a rotary vacuum evaporator. The concentrated extract was then evaporated on a boiling water bath until a constant weight was obtained. The dried extract was weighed and the yield was calculated. The extract was kept in air tight container protected from light until used.

### 2.3. Determination of Total Flavonoids Content

Total flavonoids were analyzed using aluminum chloride colorimetric method. Sample (500 *μ*g/mL) of 500 *μ*L was mixed with 500 *μ*L of 2% aluminum chloride solution. The mixture was allowed to stand at room temperature (28 ± 2°C) for 10 min with intermittent shaking. The absorbance of the mixture was measured at 415 nm against a blank sample (methanol) without aluminum chloride using a UV-vis spectrophotometer (PerkinElmer, USA). The total flavonoids content was determined using a standard curve of quercetin (0.5–12.5 *μ*g/mL). The content was calculated as mean ± SD (*n* = 3) and expressed as milligrams of quercetin equivalents (QE) in 1 g of the extract and dried powder.

### 2.4. Determination of Total Phenolic Compounds Content [13]

The content of total phenolic compounds was determined using Folin-Ciocalteu procedure. The sample (250 *μ*g/mL) or standard gallic acid solution (10–100 *μ*g/mL), 0.2 mL was mixed with 0.5 mL of the Folin-Ciocalteu reagent (diluted 1 : 10 with deionized water) and 0.8 mL of sodium bicarbonate solution (7.5% w/v). The mixture was allowed to stand at room temperature (28 ± 2°C) for 30 min with intermittent shaking. The absorbance of the mixture was measured at 765 nm using a UV-vis spectrophotometer (PerkinElmer, USA). The content of total phenolic compounds was calculated as mean ± SD (*n* = 3) and expressed as milligrams of gallic acid equivalent (GAE) in 1 g of the extract and dried powder.

### 2.5. Animals

Twenty male* (Mus musculus*) ICR mice (25–35 g), 5 weeks of age, were obtained from the National Laboratory Animal Centre, Mahidol University, Salaya, Nakhon Pathom, Thailand. The animals were habituated to the laboratory animal room for at least 1 week before the experiment. They were housed in groups of four or five animals in standard cages containing a supply of pellet diet and ad libitum water. The animal room was maintained at 25 ± 2°C with constant humidity (65%) and a 12 h of dark-light cycle. All studies were conducted between 9 a.m. and 1 p.m. The experiment was approved by the Institutional Animal Care and Use Committee, Faculty of Pharmacy, Mahidol University, Bangkok, Thailand (Proof no. PYR002/2554).

#### 2.5.1. Drugs

Diazepam (Roche, Italy) was obtained from Yangon General Hospital in a tablet form. It was dissolved in distilled water to the concentration of 0.2 mg/10 mL. Sodium pentobarbital was purchased from Sigma-Aldrich (USA), dissolved in 0.9% normal saline, and adjusted to the concentration of 50 mg/10 mL.

#### 2.5.2. Pentobarbital-Induced Sleeping in Mice

Mice were randomly divided into 4 groups of 5 mice each and treated as follows: group 1 was orally administered with a single dose of distilled water (10 mL/kg body wt.) as the normal control group; groups 2 and 3 were orally administered with the single dose of 50% ethanolic extract from ZMS at 100 and 200 mg/kg, respectively. Group 4 was the positive control group, which was orally treated with diazepam (0.2 mg/kg). Pentobarbital-induced sleeping effect was performed as previously reported [14]. Briefly, the animals were orally administered with distilled water, the ZMS extracts, or diazepam. Thirty minutes later, sodium pentobarbital at the doses 50 mg/kg was intraperitoneally injected into each mouse to induce sleeping effect. Mice that remained immobile for more than 30 min were judged to be asleep. The interval between loss and recovery of righting reflex was used as index of hypnotic effect. The animals were observed constantly and the time of awakening as characterized by righting of animals was noted.

#### 2.5.3. Statistical Analysis

The results are expressed as mean ± standard error of the mean (SEM). The values were compared using the one-way analysis of variance (ANOVA) followed by Tukey's post hoc test for multiple comparisons. *P* values less than 0.05 were considered to be statistically significant.

## 3. Results and Discussion

**Figure 1:**
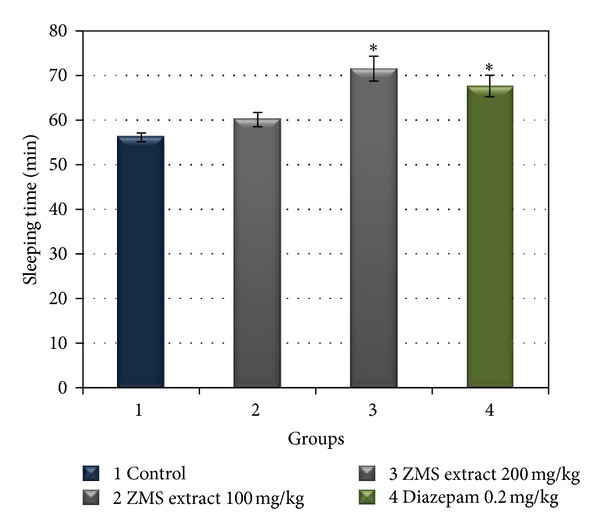
Effects of *Z. mauritiana* seed extracts (ZMS, 100 and 200 mg/kg) or diazepam (0.2 mg/kg) on sleeping time in mice induced by sodium pentobarbital (*n* = 5). All values represent mean ± SEM, *P < 0.05 compared with distilled water treated control group.

Yield, total flavonoids, and total phenolics contents in the 50% ethanolic extract of ZMS are shown in Table 1. Fifty percent ethanol extract, which was previously reported to be the appropriate extract providing high *in vitro* antioxidant activity, was used for testing the hypnotic effect in this experiment. As shown in Figure 1, the sleep duration induced by 50 mg/kg sodium pentobarbital in the control group was 56.20 ± 3.35 min. The ZMS extract potentiated pentobarbital-induced sleeping behaviors in mice in a dose dependent manner. ZMS extract at doses of 100 and 200 mg/kg significantly prolonged the sleeping time (60.25 ± 1.44 and 71.60 ± 2.72 min, *P* < 0.05 versus control group). The hypnotic effect of ZMS extract (200 mg/kg) was comparable to those of diazepam (0.2 mg/kg). Sodium pentobarbital is a drug in barbiturate group that can induce the sleep in both rodents and humans [15]. It is well known that many drugs such as benzodiazepines and sodium pentobarbital possess anxiolytic and sedative effects [16]. The classic method of pentobarbital-induced sleeping in mice is often used to screen sedative-hypnotic drugs. Previous pharmacological studies reported that ZMS reduced locomotor activities in animals [17]. The present study showed that the 50% ethanolic extract of ZMS (200 mg/kg) as well as diazepam (0.2 mg/kg) significantly increased sleeping times in mice from 56.20 ± 3.35 min in control group to 71.60 ± 2.72 and 67.60 ± 2.40 min, respectively. Our results demonstrated for the first time that ZMS extract potentiated pentobarbital-induced sleeping behavior in mice. This supports the traditional use of ZMS for insomnia and anxiety treatments [1]. The mechanism of hypnotic effect of ZMS extract may be similar to diazepam, so the further study should be investigated by using benzodiazepine antagonist drug to see a reversal sleeping time.

The hypnotic activity of herbal medicines has been attributed to different phytochemical compounds such as flavonoids, terpenes, and saponins. The active sedative components in *Z. jujuba* seeds were reported to be flavonoids and saponins [18]. Therefore, the active sedative components in the seeds of *Z. mauritiana* should be further identified and quantitatively analysed.

## 4. Conclusion

This study demonstrated the hypnotic effect of the ethanolic extract from *Z. mauritiana* seeds. The standardized extract might be developed as an alternative sedative/hypnotic product. The major active components for hypnotic effect in ZMS should be separated and identified.

## Figures and Tables

**Table 1:** Yield, total flavonoids, and total phenolics contents of 50% ethanolic extract from *Z. mauritiana* seeds.

Yield of crude extract (% dry weight)	Total flavonoids content (mg QE/g)		Total phenolics content (mg GAE/g)	
	In extract	In dried seeds	In extract	In dried seeds
30.20 ± 0.02	0.74 ± 0.03	0.22 ± 0.01	27.62 ± 1.43	8.34 ± 0.43
